# Mechanical Dentistry

**Published:** 1851-01

**Authors:** T. L. Buckingham

**Affiliations:** Dentist, Philadelphia.


### SELECTED ARTICLES.

### ARTICLE XII.

*Mechanical Dentistry.* By T. L. Buckingham, Dentist, Philadelphia.

*Taking an Impression in Wax.*—Select a cup large enough to leave a space of an eighth of an inch for the wax between the cup and the gum. Prepare the wax by softening before a fire, and working it in the fingers until it is soft enough. Fill the cup full of wax, giving it somewhat the shape of the gum, and using no more than is necessary. Put it in the mouth, standing behind the patient when the impression is for the upper jaw, and before when it is for the lower jaw. Put one hand on each side of the cup, and press it gradually up until the impression is nearly deep enough; then, holding the cup steady with one hand, press the wax close to the gum on the outside, and also to the roof of the mouth; now put one hand on each side of the cup, and press it up a little farther, and the impression is complete. To remove it, take hold of the handle of the cup, and loosen it gently, and take it out of the mouth by turning it so that one side will come out first. Be careful not to let the lips bend the edge of the wax over into the impression. If, in loosening the wax from the mouth, the teeth have drawn the wax up around the impression of them, it can be cut off with a knife. Compare the impression with the mouth, to see if it be correct, and fix in your mind the teeth that are to be clasped, if clasps are necessary.

*Taking an Impression in Plaster.*—Plaster impressions are taken of the upper jaw only, and where there are no teeth; for, if there are teeth, they would break the impression so much in removing it, that it would be useless.* The cup should be of the shape of a suction plate. If such a one is not to be had, an ordinary cup can be substituted, by building it up back with wax, to keep the plaster from running out. Make it as near the shape of the mouth as possible, only larger. Mix the plaster pretty thick, stirring it well while mixing, and pour into the cup, leaving it stand a moment, or until it becomes so stiff that it will build up without settling down. The patient should have a napkin spread over the breast to protect the dress from any plaster that might fall, with the head inclined forwards to prevent the possibility of any of the plaster going down the throat.. Get the plaster into the mouth as gently as possible, and press the cup gradually up until the impression is deep enough, and hold it there until the plaster sets, which will be from three to five minutes.† It can be told when the plaster is

* There are some who succeed in taking an impression of the lower jaw in plaster; and also in taking impressions where there are teeth remaining in the mouth; the latter they accomplish by breaking the impression in the mouth, and connecting the pieces afterwards by cement.

† The plaster will harden sooner by mixing it with salt water. This will do in taking impressions, but will not answer for casts, as it makes the plaster crumble.

hard enough, by trying what hangs over the edge of the cup, or what has been left in the vessel the plaster was mixed in. When it breaks short, or has lost the sticky feel, it will do to remove it from the mouth.

Plaster takes a much better impression than wax. The wax, when pressed up, will slide over the roof of the mouth, which often spoils the impression; and again, it often sticks to the mouth, and is difficult to remove, or the edges will be curled over in removing it from the mouth; but none of these objections will apply to plaster, excepting that the edges may be broken in removing it; but these can be replaced and made perfect by using a little cement.

I am satisfied that if the mode of taking impressions with plaster be once adopted, it will not be abandoned, at least not to go back to wax. Out of a number of cases, I have never failed to make a good fit when I took the impression in plaster.

*To make the Plaster Cast.*—Take a strip of sheet lead, such as is used for cutting patterns, or a piece of paper folded three or four times, leaving it some two inches wide, and a foot or more long. Set the wax impression after oiling it, on the table, and fold the strip of paper around it, and so shape it that the cast will be largest at top;* mix the plaster just thick enough to flow into all the indentations in the impression; then with a knife or spoon, drop a small quantity into the impression, commencing at the roof of the mouth, letting it flow down gently, filling up the impressions of the teeth, when the balance may be poured in from the cup until you have the cast from one and a half to two inches thick. Let it stand until the plaster is hard enough to keep its form, then remove the band and let it remain until perfectly hard. The wax can be removed by first cutting it away, so as to free the edge of the cup all around, then by running the point of a knife between the cup and wax, the cup can be forced away, leaving the wax on the cast. Then hold the wax to the fire until it is quite soft, and holding the

* In taking a cast from a plaster impression, allow the impression to become perfectly dry, then oil it, and cast as described for wax.

cast in one hand, bend the wax up in front until the surfaces of the teeth are exposed, then cut the wax away until the teeth are all free, then the large portion of wax in the cavity of the cast can be removed in a lump. The cast should now be trimmed, so as to draw readily from the sand. If the front of the cast, or the teeth on one side should project, there must be left a corresponding fulness on the opposite side; or, that a line drawn lengthways with the teeth in front, and one drawn on the opposite side of the cast would diverge as they go from the teeth to the top of the cast.

If the cast is not perfect around the teeth, it must be trimmed until it is so. Of course, the object is, to have a perfect model of the mouth. It is well to varnish the cast with some spirit varnish; I prefer a varnish made by dissolving gum sandrach in alcohol, as it leaves the cast almost white, and penetrates farther into the cast, thereby making it harder than the shellac varnish, which is generally used.

If the points or edges of the teeth are closer together than their necks, the space should be filled up with wax until the sides are at least parallel. If it is designed to fit a cavity plate, the wax for forming the cavity should be put in after the cast has been varnished, as it sticks much better on the varnished cast; and it can be varnished also.

*Moulding in Sand.*—Take some of the finest casting sand,* just moist enough to hold together; fill a small vessel, say a common tin cup. If it is too wet, the hot metal when poured into it, will cause so rapid an evaporation of the moisture as to make the metal boil; and if too dry, will crumble when the metal is poured in, either of which would spoil the cast. Screw an ordinary stump screw into the top of the cast, and press the cast, teeth down, into the sand, then pack the sand around the cast until the sand is level with the top of the cast; now take hold of the screw with one hand, and with a small hammer in the other, tap the shank of the screw gently, so as to jar the

* We have noticed that common whiting has been used instead of sand, with good success.

cast in the sand, and turning the cup around, striking the screw on several sides, until the cast is loose, then lift it up slowly and carefully, tapping the screw in the meanwhile, until the cast is out of the sand. If the sand has drawn up around the teeth, brush the sand off the cast, and replace it in the mould, going through the operation as before, and if necessary, repeat, until the mould is perfect. If a little loose sand should fall into the mould, it can be turned up and the sand blown out. The sand should not be packed too tight, but have it sufficiently porous to allow the vapor to pass through it.

*Making the Zinc Cast.*—Melt a sufficiency of zinc, and when it has just melted take an old knife, or something similar, and skim off all the dross; let it stand until it begins to harden, or stick to the sides of the ladle, then pour it into the mould, beginning at a part of the mould where it is not required to be perfect, for fear of the metal washing the sand, continue to pour gently, with the ladle close to the sand, until the mould is full. Let it stand till the metal is hard, then remove it and cool in water. If the cast is not perfect around the teeth, the superabundance can be cut away with a coarse file and graver; and, if necessary to separate the teeth on the zinc cast, it can be done with a small saw, such as carpenters use for sawing circles. The zinc cast should, of course, be a fac-simile of the plaster model.

If a zinc cast has been properly made, it will be as bright and smooth as a piece of silver plate. If the zinc be poured too hot it will boil, though the sand be in a proper state.

*To make the Lead Cast.*—Put the zinc cast into a cup or ring, with the teeth up, and with a small quantity of sand under it, to make it set steady; then fill the cup with sand, and pack it down, until it is three-quarters of an inch above the teeth, then cut the sand out with a knife, until all the parts of the zinc cast, where the plate is to fit, are exposed. Where the plate curls over the gum, the lead cast should be thick, but it need not run farther down on the zinc cast than the plate is to go. If the lead cast be too deep, it makes it more difficult to swage the plate, and the plate will stick in the lead, and be bent in getting it out. Melt the lead in another vessel than the one in which the zinc was melted, for if but a small portion of lead be mixed with the zinc, it will spoil it for future use. Pour the melted lead over the zinc cast as prepared, and in a few minutes remove them and wash clean, and knock them apart with a hammer, and all is ready.

*To make a Plate.*—Take some thin sheet lead, and by pressing it down on the cast and marking and cutting out, get a pattern of the size the plate is desired, then spread the pattern out, and lay it on the plate, and mark and cut out; anneal the plate by heating; now, with a pair of pliers, or bending plate forceps, and a small wooden mallet of suitable size, fit the plate as near as possible, to the zinc cast, then put your casts together with the plate between them, and strike the zinc cast lightly with a hammer, then take them apart and see if the plate is in its proper position, and repeat, striking the cast a little harder, until the plate is nearly up, when, with two or three smart blows, the plate is made to fit the cast accurately.

In swaging a plate be sure to get it started aright, and it may be necessary to anneal it two or three times during the operation, and also to file away a little if it should bind too tightly in any place. When the plate fits the zinc cast, it should be tried on the plaster cast to see if it fits it also. Then bend the clasps with a pair of round-nosed plyers to fit the teeth as near as possible, particularly around the necks of the teeth where they are to be soldered to the plate, and file the plate away to allow them to go down between it and the teeth; now arrange the plate and clasps on the plaster cast, and stick them together with some wax or cement, then lift them carefully from the cast, and set them on a piece of charcoal, then pour some mixed plaster over the ends of the clasps and under the plate; when it gets hard, remove the wax, and if there is any place where the clasps stand off from the plate, fill it up with scraps of gold; now coat the places or joints, where the solder is to flow, with borax, and lay on the solder and melt or flow it; when it is cold, try it on the zinc cast, and fit the clasps up to the teeth with a small hammer, then remove and file the clasps and plate to the shape they are to be, then put the plate on the zinc cast, and, after cutting away the lead cast so as not to drive the clasps down too far, put them together and swage the plate up again, and see that it fits the plaster cast. The plate should now be boiled in some diluted sulphuric acid, for a few minutes, to remove the fire coat, and, after washing it, stone up and polish the plate. Some do not polish till after the teeth are soldered on, but I think much time is saved by polishing at this stage of the operation, as it can be done so much more quickly than when the teeth are on. Now try the plate in the mouth and make it fit perfectly easy. Cut, file and bend it if required, till both yourself and patient are satisfied.

It is very important that we should have the confidence of the patient, and this can generally be secured by giving satisfaction, and also by making all necessary explanations.

When the plate has been properly adjusted, take the close of the mouth, as follows: Put the plate on the plaster cast, and arrange the wax on the plate where the artificial teeth are to go, leave the wax longer and fuller than the artificial teeth are to be, then put the plate in the mouth with the wax on it, and have the patient close the mouth naturally, and if there are natural teeth in the opposite jaw which antagonise with teeth in the jaw you are fitting, let the mouth be closed until they come together. If there should be no natural teeth to articulate, some hard substance had better be put into the wax to keep the patient from biting too far into it. Notice, that in closing the mouth, the under jaw has not been projected or twisted laterally, but that it has been a natural close. As patients are very apt to project the lower jaw in closing the mouth, a good plan to prevent it is, to put the hand against the lower jaw, and press backwards as the jaw closes. If the close has been correct, mark the exact centre of the mouth, and remove the plate and wax, and select a tooth for shade.

*To make an Articulating Cast.*—Place the wax with which the close of the mouth was taken and plate, on the plaster cast, and fill up the roof of the mouth even with the points of the teeth, and nearly as far back as the back of the cast; then cut a V shaped groove down the back of the cast, and oil it well, so that the articulator will not stick to it; then mix some plaster and drop it into the impressions of the under teeth, and let it flow down the back of the cast, filling up the groove; then make the plaster thick enough to build up with a knife.

The articulator should be about half an inch thick on the top and back. When the plaster has set, it can be trimmed off, and the casts taken apart. When the plate and wax are removed, and the casts put together, we have a fac-simile of the mouth when closed.

The teeth may now be selected to suit the case; the shade tooth and articulating cast will be sufficient guides in their selection. To grind the teeth to the plate a grinding machine of some kind is necessary, with several sizes of emery wheels. I prefer a small lathe, as it best answers to polish plates. Put the plate on the cast, and commence to grind the teeth to fit. If it is intended to run the necks of the teeth over the plate and press upon the gum, the cast should be scraped away about the thickness of a five cent piece; if, however, the gum be very soft or spongy, it may be scraped even further. The teeth should be ground to fit the plate and the neck-press upon the cast, allowing sufficient projection to clear the under teeth. The necessary projection and length will be ascertained by putting the casts together, as the teeth are ground to suit, they must be attached to the plate in their proper position with cement. (Cement may be made by melting beeswax and resin together.)

Another and quicker way to grind the teeth, is to build up some wax on the plate, and arrange the teeth around on the wax, but as we cannot see the back of the teeth for the wax, we have to grind by guess, and often leave a space between the base of the teeth and plate.

When the teeth have been ground and properly arranged, remove the plate and teeth from the cast, and set them on a piece of charcoal, then mix some plaster and sand, about equal parts, and pour it around the teeth, and let it flow under the plate; when the plaster is about half way up the front of the teeth, put a piece, or two or three pieces, of stiff iron wire, twisted together, around the front of the teeth, so that if the plaster should break in heating, the teeth will not be drawn out of place ; then build up the plaster until the points of the teeth and the clasps are covered. The plaster should be about half an inch thick. If there be too much plaster, it will take an extra heat to solder the teeth, and if too little, it is liable to break before the teeth are soldered. When the plaster has become hard, the cement that held the teeth to the plate can be removed, and the stays put on the teeth.

In putting the stays or lining on the teeth, take a strip of gold —if a gold case—of suitable width, or nearly as wide as the teeth, and stand it up against and along the back of the tooth, and file the end, if necessary, to fit the curve of the plate to which the lining is to be attached when soldered, move it sideways against the lower pin of the tooth, and it will be marked where the pin comes, then with the punch forceps, punch a hole for the pin, then put it back again with the pin in hole, and move the strip as before against the upper pin, by which you get the spot to punch the other hole ; see that it fits the tooth after filing off the metal which the punch has driven through, then countersink the holes on the outer surface, and cut the lining off the proper length. I generally leave the lining large enough to cover nearly the whole surface of the tooth. In punching the linings, punch always from the side that goes next the tooth.—*Dental News Letter.*

